# State-of-the-art 3D analysis of soft tissue prediction for orthognathic correction of dentofacial deformities in the Egyptian population

**DOI:** 10.1186/s12903-026-08833-2

**Published:** 2026-06-30

**Authors:** Nehal Ibrahim Shobair, Xiangyang Ju, Amr Amin Ghanem, Mohamed Diaa Zain El Abedeen, Amr Ekram, Ahmed Abdel Moneim Barakat, Ashraf Farouk Ayoub

**Affiliations:** 1 Department of Oral and Maxillofacial Surgery, Faculty of Dentistry, Ain Shams University, Cairo, Egypt; 2 Medical Devices Unit, NHS Greater Glasgow and Clyde, Glasgow, UK; 3 Department of Oral and Maxillofacial Surgery, Faculty of Dentistry, Cairo University, Cairo, Egypt; 4 School of Medicine, Dentistry and Nursing, University of Glasgow, Glasgow Dental Hospital and School, Glasgow, UK

**Keywords:** Orthognathic surgery, Three-dimensional imaging, Soft tissue, Computer simulation, Treatment outcome, Prognathism, Cone-beam computed tomography

## Abstract

**Objectives:**

To evaluate the accuracy of soft tissue simulation in bimaxillary osteotomy for orthognathic surgical correction of Class III dentofacial deformities using 3D conformation meshes and facial aesthetic units.

**Materials & methods:**

A retrospective analysis of CBCT scans of 13 Egyptian patients who underwent simultaneous maxillary advancement and mandibular setback surgery for correction of class III skeletal deformities. Preoperative and postoperative CBCT scans were superimposed using voxel-based registration. Dolphin software generated soft tissue predictions according to the measured surgical movements. This was compared with the actual soft tissue changes following orthognathic surgery. Prediction accuracy was assessed using dense surface correspondence analysis with region-specific segmentation into facial aesthetic units, comparing Euclidean and directional (X, Y, Z) distances between predicted and actual outcomes, and color-coded maps were used to visualize the findings.

**Results:**

Across all regions, the highest prediction errors was in the anteroposterior dimension, with the upper lip showing the greatest deviation (median 2.04 mm). The chin region exhibited the lowest overall prediction error of 1.82 mm. The lower lip and cheek regions showed moderate prediction errors. The prediction error was relatively low at the nose. Color maps indicated a consistent pattern of underprediction of the soft tissue changes following Le Fort I maxillary advancement and mandibular setback surgery.

**Conclusion:**

Prediction errors across facial regions remained within an acceptable range of less than 2 mm. The sophisticated methodology of this study, incorporating dense correspondence analysis and facial unit segmentation, provided a more precise assessment of soft tissue prediction accuracy of bimaxillary osteotomy for correction of class III deformities. These findings support the clinical reliability of Dolphin software while highlighting the importance of advanced analytical approaches in orthognathic surgical planning.

**Clinical relevance:**

The application of the conformation meshes and the mathematical facial segmentation into aesthetic units provided an insight into the magnitude and direction of 3D prediction errors in Egyptian population, this should be considered in planning orthognathic surgery.

## Introduction

The main objective of orthognathic surgery is the correction of functional impairments and address aesthetic concerns associated with dentofacial deformities. Surgical procedures should be planned to improve dental occlusion facial balance, and positively affecting the quality of life. Skeletal Class III malocclusion is a common deformity that requires careful surgical planning for the coorection of maxillary deficiency or mandibular prognathism or both. The accuracy of surgical planning is pivotal in achieving the desired outcomes, particularly for the soft tissue changes, which significantly influence postoperative aesthetics and patient satisfaction [1, 2].

Various methods and software packages have been developed to enhance three-dimensional (3D) soft tissue prediction in orthognathic surgery. These include Dolphin 3D, ProPlan CMF, IPS (KLS Martin), and SurgiCase [3–6]. While all are widely used, the mathematical basis of soft tissue prediction differs markedly among the packages.

Several approaches have been developed to analyze the prediction accuracy of soft tissue changes in orthognathic surgery. Landmark-based analysis is one of the simplest approaches for the evaluation of 3D facial images. It is based on manual or semi-automatic identification of a set of anatomical points and a limited number of landmarks. This allows the point correspondence analysis to be carried out but fails to represent the complex three-dimensional facial morphology [6]. Surface-based mesh analysis was introduced to overcome this limitation [7] The conventional generic meshes have been used for this purpose, but they lack true anatomical represenation of facial regions. This approach effectively addresses many of the limitations of landmark-based methods but requires sophisticated algorithms and greater computational resources. Attempts to improve this approach by excluding stable regions and dividing facial meshes into anatomical areas improved regional description but did not resolve the absence of vertex-to-vertex anatomical correspondence. Therefore, in this study, we conformed the genric mesh to the 3D facial morphology, and the confomed mesh was used in the analysis.

There is a critical need to investigate the accuracy of the 3D prediction of existing software packages for orthognathic surgical planning. Ideally, 3D prediction planning should be sensitive enough to quantify subtle changes in facial morphology, including both magnitude and directionality, to support clinical decision-making [7]. In the present study, dense correspondence analysis combined with generic mesh conformation preserves anatomical correspondence across the entire facial surface, enabling robust region-specific and direction-specific evaluation of soft tissue prediction accuracy.

The aim of this study was to evaluate the accuracy of soft tissue prediction following bimaxillary orthognathic surgery for correction of Class III skeletal deformities. We also aimed to assess the reliability of the dense surface correspondence method for analyzing soft tissue prediction accuracy in orthognathic surgery. The rationale of this study is to improve the quality of patient care and achieve predictable soft tissue outcomes following orthognathic surgery.

## Methods

This retrospective study analyzed the facial CBCT scans of 13 orthognathic patients treated at Ain Shams University Dental Hospital and School, Cairo, Egypt. All patients were of the same ethnicity (“Egyptians”) and diagnosed with Class III dentofacial deformity based on clinical examination and standard cephalometric parameters, including maxillary hypoplasia and mandibular prognathism. All patients underwent bimaxillary osteotomy, Le Fort I maxillary advancement and sagittal split mandibular setback. Ethical approval was obtained (Reference Number: FDASU-Rec ER032506), and the study was registered at ClinicalTrials.gov (NCT06893614).

Sample size calculation was performed using G*Power version 3.1.9.2, targeting 90% accuracy based on Resnick et al. (2016) [6]. The computed effect size for linear measurement accuracy was 1.05, with α = 5% and β = 20% (power = 80%), resulting in inclusion of 13 subjects. Patients agre range was 17–35 years. To reduce variability related to ethnic differences in facial soft tissue morphology, only Egyptian patients were included in the study. Diagnosis of skeletal Class III malocclusion was established using combined clinical and cephalometric criteria, including concave facial profile, negative overjet, and confirmation of sagittal discrepancy (ANB angle and Wit’s appraisal). All cases required bimaxillary orthognathic correction based on comprehensive orthodontic and surgical evaluation. Exclusion criteria included craniofacial anomalies (e.g., cleft lip/palate), previous maxillofacial surgery, segmental Le Fort I osteotomies, and cases with simultaneous genioplasty or malar augmentation. In all the cases surgical planning was performed using a standardized virtual workflow.

**Fig. 1:**
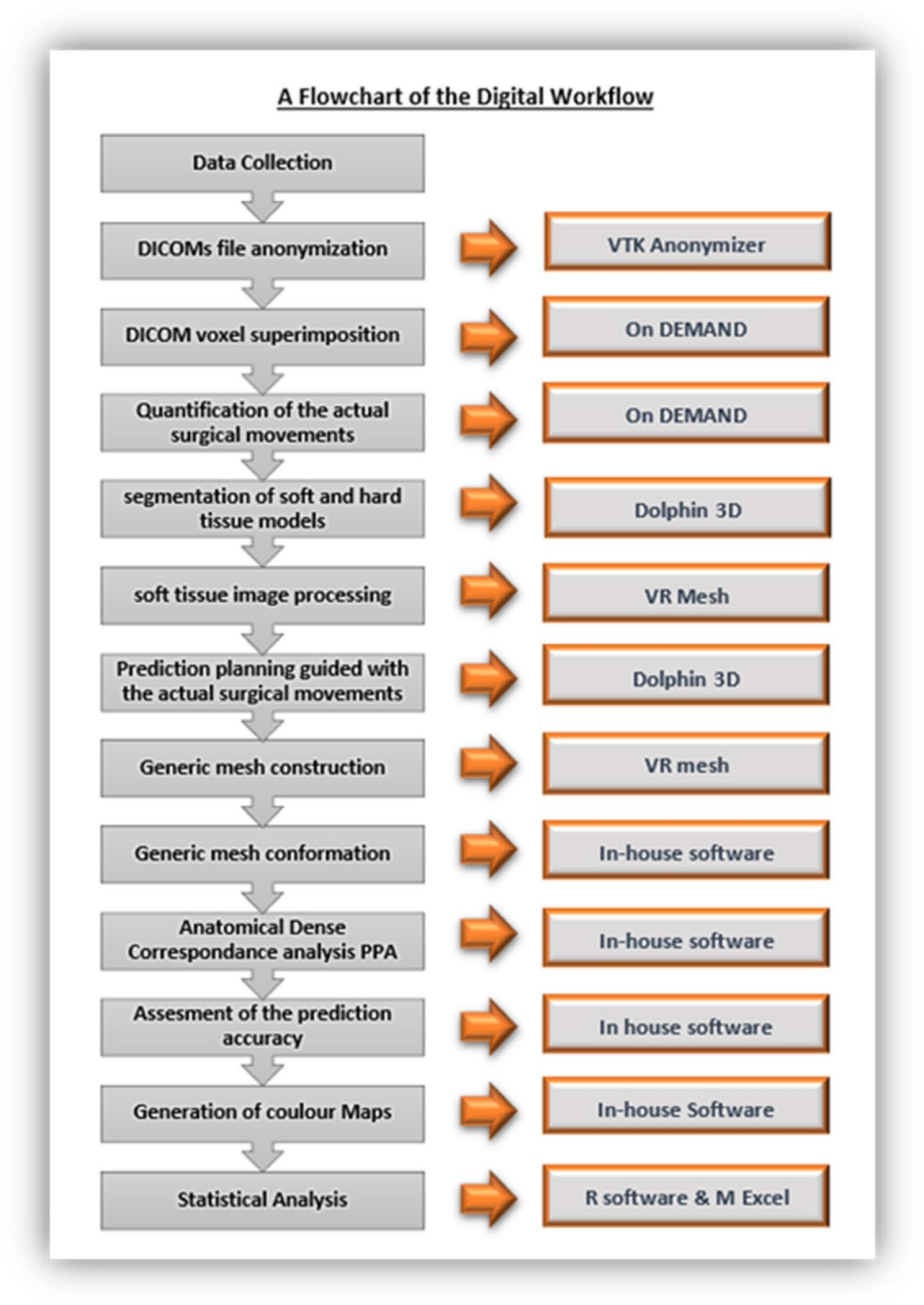
A Flowchart illustrating the digital workflow of the case processing, detailing the software utilized at each stage

Preoperative CBCT scans for 3D prediction planning were acquired within one month before surgery, and postoperative scans were obtained 6–12 months after surgery. Analysis of skeletal and soft tissue changes followed a structured digital workflow (Fig. 1). Pre- and postoperative scans were imported into OnDemand3D software (Cybermed, Seoul, South Korea), with the preoperative scan designated as the reference image for superimposition.

The magnitude and direction of achieved surgical movements were measured as described by Almukhtar et al. (2015) [8]. Soft tissue predictions were generated based on these movements and compared with actual postoperative changes.

### Analysis of the accuracy of soft tissue prediction

A 3D mathematical facial mask (generic mesh) consisting of approximately 7,000 vertices with known x, y, and z coordinates was used. The X-axis represented mediolateral (left–right), the Y-axis vertical (superior–inferior), and the Z-axis anteroposterior (front–back) directions. The generic mesh was conformed to the 3D facial surface using non-rigid registration to establish anatomically indexed vertex correspondence. The conformed mesh was derived from CBCT scans, extending from the hairline to the submental region and bilaterally to the tragus. The conformation algorithm was developed at the University of Glasgow Dental Hospital and School, UK.

**Fig. 2:**
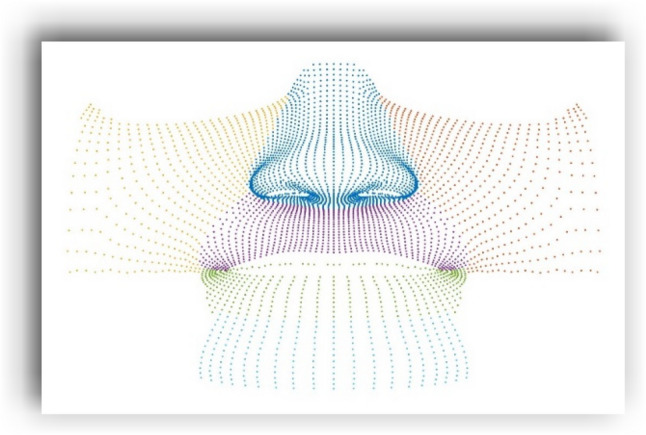
Generic facial mesh and segmentation into six anatomical regions prior to conformation, forming the basis for dense correspondence analysis. The aesthetic units are Nose, upper lip, lower lip, right cheek, left cheek, chin

Using VRMesh software, the generic mesh was duplicated and segmented into six anatomical regions: nose, left and right cheeks, upper lip, lower lip, and chin. The accuracy of this conformation process has been validated by Cheung et al. (2016), demonstrating sub-millimeter median Euclidean error [9] (Fig. 2). Segmentation preserved original vertex indices to ensure analytical consistency. The conformation process followed Ju and Siebert (2001) [10] and included global mapping and local deformation stages. In global mapping, corresponding anatomical feature points were manually identified on both the generic mesh and scanned surface. We applied a novel three-dimensional mapping function that portrayed the 3D anatomical characteristics of facial morphology. This is of a particular importance in this study for the assessment of the accuracy of 3D prediction planning at the selected anatomical regions. A generic mesh is a universally applicable facial surface "a mathematical facial mask" representing morphological information of an average face, which consists of common morphological characteristics within the population. This generic mesh was elastically deformed in a process known as mesh conformation to represent the patients/participants underlying facial morphology, thus creating a conformed mesh specific to each patient. This mapping was achieved using radial basis function (RBF) interpolation:

$$f\left(\mathbf{t}\right)={a}_{0}+{a}_{1}{t}_{x}+{a}_{2}{t}_{y}+{a}_{3}{t}_{z}+\sum\:_{j=1}^{n}{b}_{j}{\hspace{0.17em}}\sigma\:(\parallel\:\mathbf{t}-{\mathbf{t}}_{j}\parallel\:),$$

where σ(r) = r is the basis function, and coefficients were determined by solving a constrained linear system. The local deformation stage refined the fit by displacing mesh polygons toward their nearest surface points using a Binary Space Partitioning (BSP) tree for efficient correspondence identification. This two-step process ensured accurate mesh adaptation while preserving topology and geometry.

**Fig. 3:**
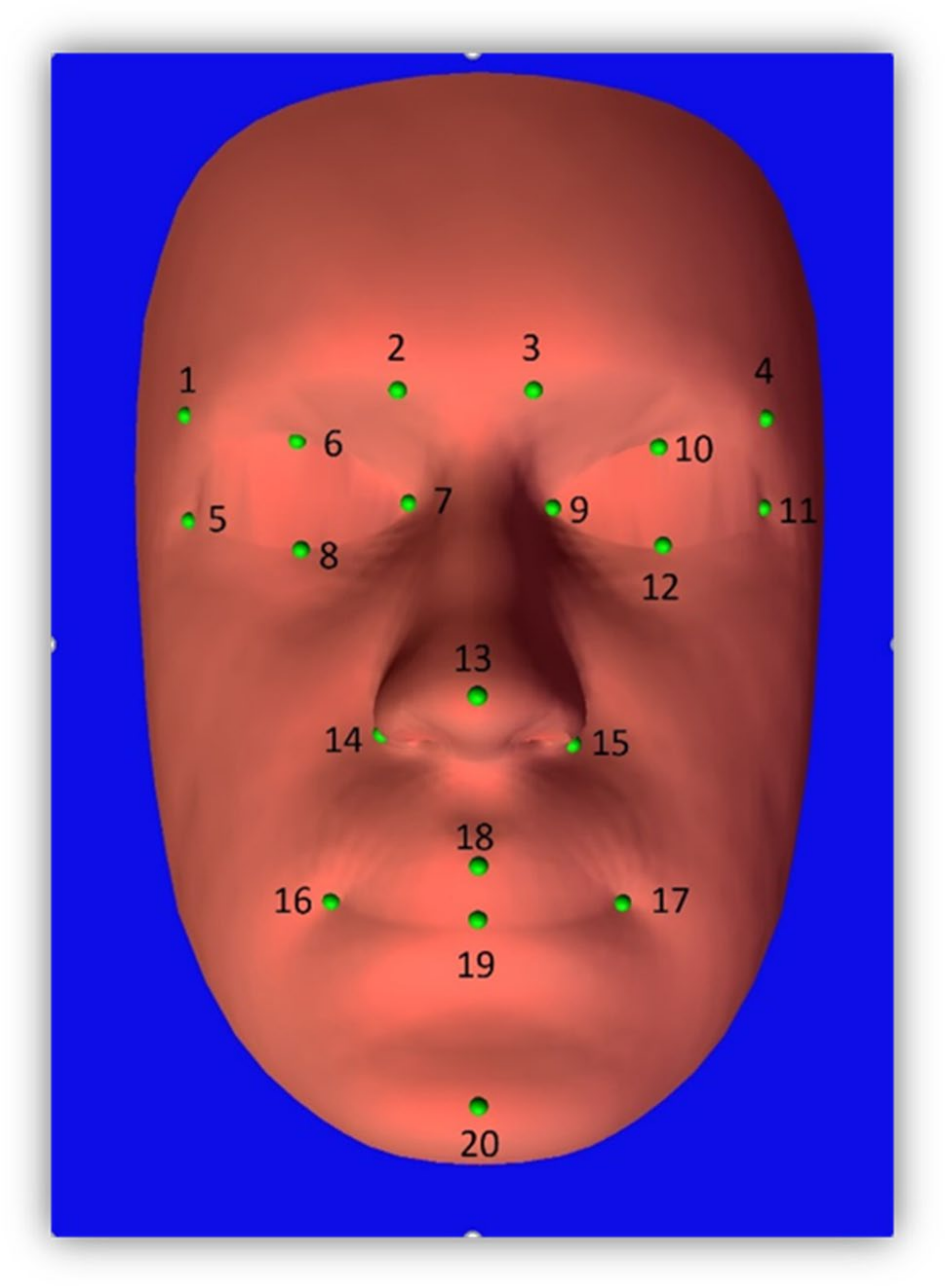
Landmark for generic mesh conformation

**Fig. 4:**
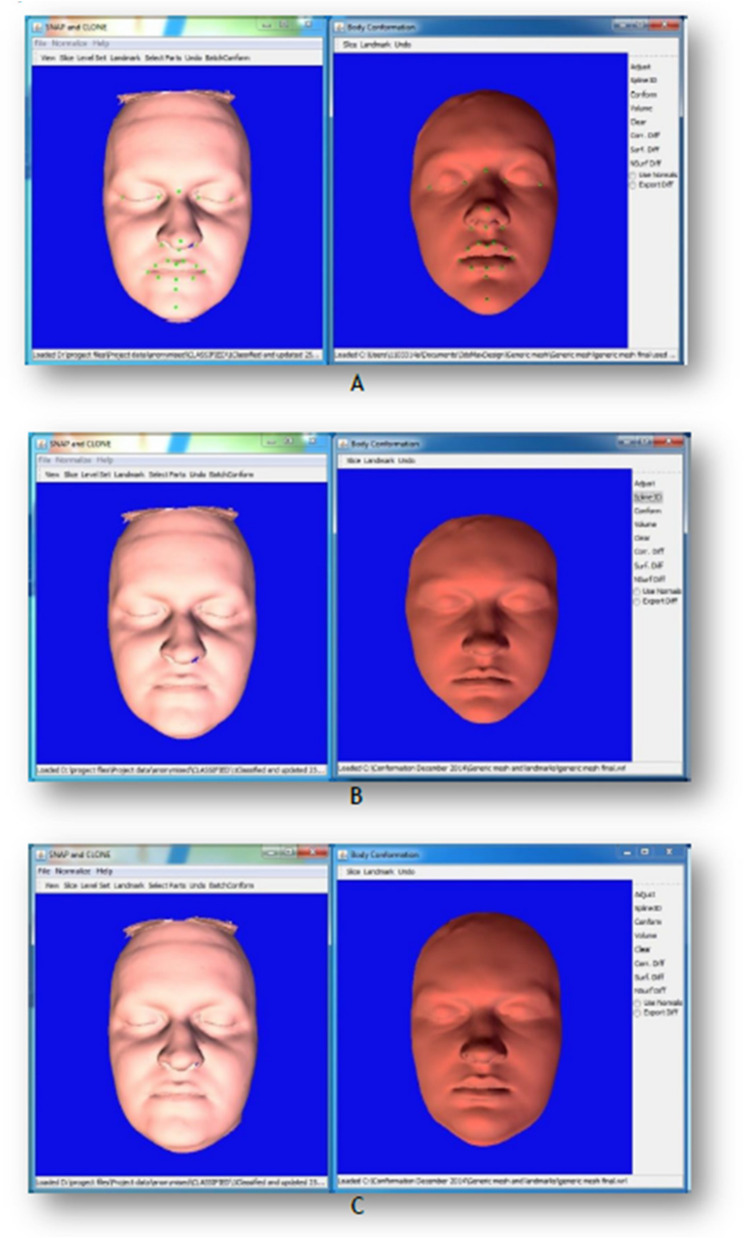
The conformation software shows the main panel with two 3D images loaded: the target image (left side) and the generic mesh (right side). It demonstrates the steps of generic mesh conformation: Landmarks placement (**A**), initial elastic deformation (**B**), final elastic deformation (**C**)

A total of 20 landmarks (Fig. 3) were digitized on both the generic mesh and on the 26 facial models (13 actual postoperative and 13 predicted 3D images). Landmark identification began semi-automatically, followed by automated conformation, whereby the mesh was elastically fitted to the CBCT-derived soft tissue surfaces (Fig. 4). The resulting conformed meshes were saved as VRML (wrl) files. This generated 26 conformed meshes representing predicted and actual postoperative morphology, these were used for the analysis of soft tissue prediction accuracy.

### Anatomical dense correspondence analysis

Differences between predicted and actual soft tissue were analyzed by calculating absolute differences across all 13 cases. For each case, key metrics were generated to assess the soft tissue prediction accuracy. Each matrix included minimum, maximum, regional mean, standard deviation (SD), absolute maximum, absolute mean, and absolute SD for 90% of points within each anatomical region. These values were averaged across six predefined facial aesthetic units and analyzed separately for each spatial dimension (x, y, and z). Results were visualized using box-and-whisker and Altman plots to illustrate median, range, and interquartile distribution across regions and dimensions.

### Assessment of landmarking error (intra-observer reliability)

Landmarks were identified by a single trained observer. The procedure was repeated after two weeks to assess intra-observer reliability and method validity. Statistical significance of positional differences between repeated landmark digitization across the three dimensions (x, y, and z) were assessed. Euclidean distances between repeated landmarks were also calculated for each point and the reproducibility of landmarking was assessed using Student’s t-test.

### Statistical analysis

Normality of data distribution was assessed using the Kolmogorov–Smirnov and Shapiro–Wilk tests. For parametric data, paired Student’s t-tests were used to evaluate differences between actual postoperative soft tissue changes and predicted values. For non-parametric data, the Wilcoxon signed-rank test was applied to compare the actual with the predicted soft tissue measurements. The significance level was set at $\:P\le\:0.05$.

**Fig. 5:**
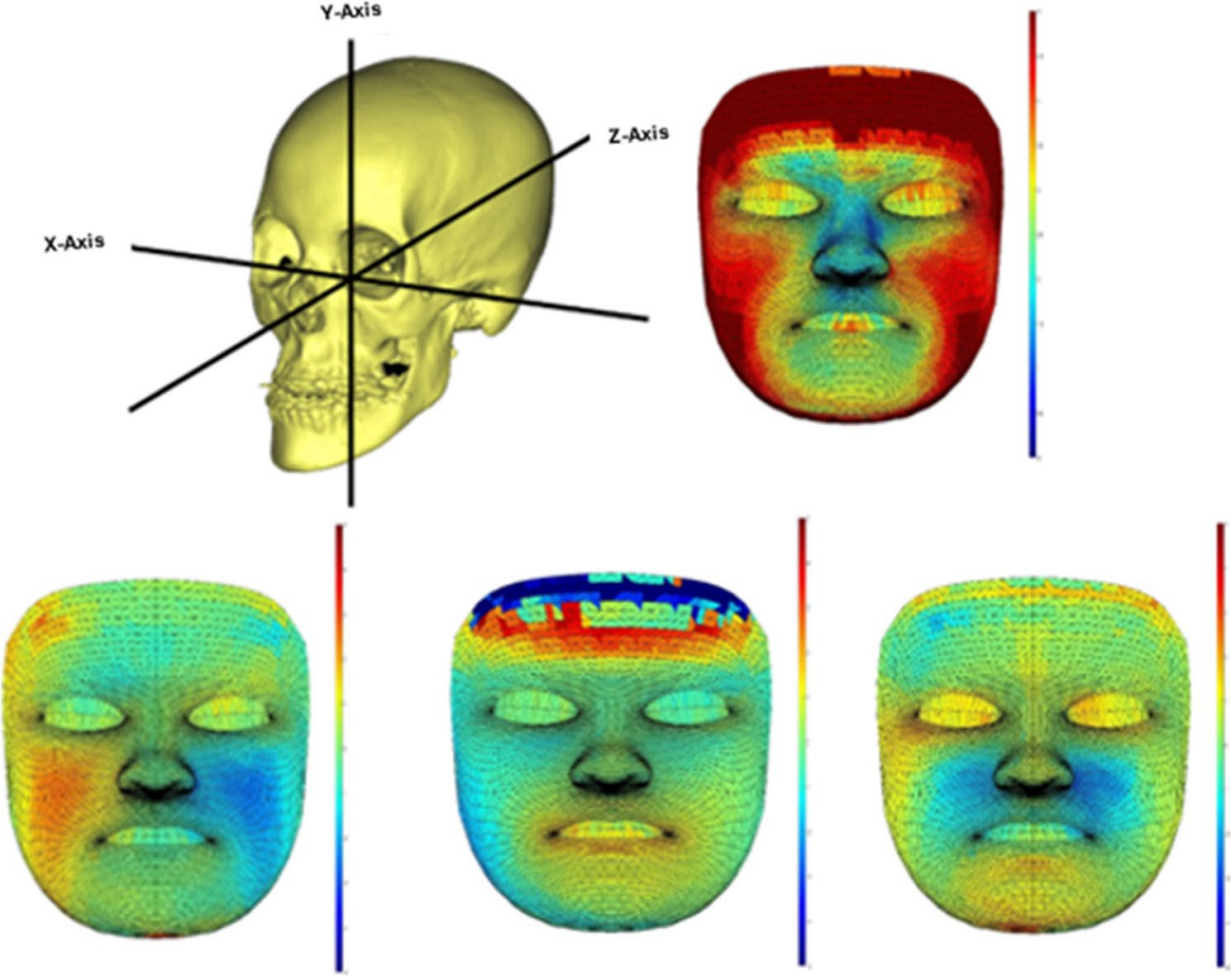
Color-coded distance maps illustrating differences between predicted and actual soft tissue surfaces..(**A**) Illustration of the 3D planes of space: X-axis (mediolateral), Y-axis (Vertical), and Z-axis (anteroposterior). (**B**) A color map displaying the Euclidean distance magnitude between the predicted and actual postoperative soft tissue changes. (**C**) A directional color map depicting the average error magnitude in the Y-dimension (vertical) for the upper lip region. (**D**) A directional color map illustrating the average error magnitude in the X-dimension (mediolateral) for the upper lip region. (**E**) A directional color map representing the average error magnitude in the Z-dimension (anteroposterior) for the upper lip region

Statistical analysis was performed using the “R” software. Statistical hypothesis testing (p$\:\le\:0.05$-) was applied to evaluate landmarking error and intra-observer reliability analyses. The descriptive regional soft-tissue prediction errors were analyzed using the median values and interquartile range (IQR). Based on the orthognathic literature, a threshold of ≤ 2 mm of prediction accuracy was considered clinically acceptable [6]. The distances between corresponding mesh vertices were measured in millimeters and visualized using a color scale: red indicates overprediction, blue demonstrates underprediction, and green indicates perfect accuracy. Color intensity reflected the magnitude of discrepancy. Directional differences were calculated along the x, y, and z axes, generating axis-specific color-coded maps (Fig. 5). This was achieved using in-house software developed at the University of Glasgow Dental Hospital and School.

## Results

### Part I: Error of the method

Table 1 shows the intra-observer landmark digitization error for the 20 landmarks used for generic mesh conformation in the x, y, and z dimensions. Three landmarks showed statistically significant errors in repeated digitization. An error of 1.8 ± 1.1 mm was noted in the x-dimension (mediolateral) for landmark 2 (medial supraorbital rim). In the y-dimension, two landmarks showed statistically significant digitization errors: landmark 12 (infraorbital rim; 0.30 ± 0.26 mm) and landmark 18 (center of the cupid’s bow; 0.47 ± 0.36 mm). Reliability of repeated landmarking was assessed using Spearman’s rank correlation coefficient (ρ), which confirmed a high correlation between repeated measurements for all landmarks in all dimensions.

**Table 1:** Intra-observer landmark digitization error showing mean orthogonal distances (± SD) in the X (mediolateral), Y (vertical), and Z (anteroposterior) dimensions, with corresponding statistical comparisons between repeated measurements

Landmark		X- Dimension						Y- Dimension						Z- Dimension					
		Mean	SD	*P*-value	ES	rs	*p*-value	Mean	SD	*P*-value	ES	rs	*p*-value	Mean	SD	*P*-value	ES	rs	*p*-value
1		1.57	1.34	0.706	0.001	0.946	< 0.001*	2	1.97	0.928	-0.001	0.965	< 0.001*	2	1.51	0.432	0.118	0.98	< 0.001*
2		1.87	1.15	0.003*	0.011	0.992	< 0.001*	0.65	0.59	0.094	0.003	0.996	< 0.001*	0.82	0.74	0.15	0.272	1	< 0.001*
3		2.42	1.11	0.071	0.01	0.979	< 0.001*	0.58	0.42	0.637	-0.001	0.979	< 0.001*	0.84	0.91	0.604	0.077	0.97	< 0.001*
4		1.81	1.52	0.755	0.002	0.975	< 0.001*	1.92	2.63	0.347	0.007	0.988	< 0.001*	0.71	0.94	0.054	0.539	0.99	< 0.001*
5		1.34	1.31	0.349	0.003	0.975	< 0.001*	1.25	1.45	0.262	-0.005	0.988	< 0.001*	0.57	0.53	0.102	0.282	1	< 0.001*
6		0.94	0.69	0.187	0.003	0.994	< 0.001*	0.67	1.18	0.511	-0.002	0.992	< 0.001*	1.12	1.63	0.325	0.046	1	< 0.001*
7		1.83	2.08	0.151	0.007	0.986	< 0.001*	1	1.27	0.724	0.001	0.992	< 0.001*	1.14	1.31	0.361	0.098	0.97	< 0.001*
8		0.67	0.55	0.884	0.0001	0.967	< 0.001*	0.38	0.38	0.648	0.001	0.998	< 0.001*	1.03	1.22	0.211	0.467	0.99	< 0.001*
9		1.37	1.99	0.080	0.009	0.994	< 0.001*	0.54	0.61	0.796	0.0001	0.994	< 0.001*	0.7	0.74	0.29	0.169	1	< 0.001*
10		0.94	0.98	0.105	0.005	0.994	< 0.001*	0.54	0.74	0.648	-0.001	0.994	< 0.001*	1.4	1.54	0.276	0.2	0.99	< 0.001*
11		1.42	1.16	0.133	0.008	0.998	< 0.001*	1.45	1.77	0.553	-0.003	0.992	< 0.001*	0.71	0.71	0.812	0.251	0.99	< 0.001*
12		0.78	0.56	0.238	0.003	0.992	< 0.001*	0.3	0.26	0.014*	0.002	0.992	< 0.001*	0.84	0.85	0.862	0.139	0.98	< 0.001*
13		0.53	0.41	0.473	0.001	0.994	< 0.001*	0.14	0.2	0.588	0.0001	0.996	< 0.001*	0.79	0.6	0.428	0.21	0.98	< 0.001*
14		0.91	0.85	0.298	0.002	0.99	< 0.001*	1.17	0.86	0.243	0.688	0.998	< 0.001*	0.69	0.59	0.927	0.735	1	< 0.001*
15		1.12	1.33	0.201	0.004	0.986	< 0.001*	0.98	0.81	0.261	0.003	0.996	< 0.001*	0.73	0.76	0.484	0.14	0.97	< 0.001*
16		1.34	1.44	0.082	0.006	0.965	< 0.001*	0.56	0.91	0.324	0.002	0.996	< 0.001*	0.75	1.05	0.385	0.197	0.99	< 0.001*
17		1.25	0.89	0.390	0.003	0.992	< 0.001*	0.75	1.11	0.115	0.004	0.992	< 0.001*	0.78	0.74	0.735	0.922	0.99	< 0.001*
18		0.92	0.66	0.605	0.001	0.994	< 0.001*	0.47	0.36	0.045*	0.673	0.998	< 0.001*	0.64	0.31	0.14	0.395	0.99	< 0.001*
19		0.9	0.73	0.761	0.001	0.992	< 0.001*	0.5	0.71	0.199	0.002	0.998	< 0.001*	0.88	1.11	0.197	0.221	0.99	< 0.001*
20		1.38	1.07	0.890	0.005	0.979	< 0.001*	0.54	0.7	0.791	0.0001	0.99	< 0.001*	2.12	2.02	0.922	0.18	0.98	< 0.001*
Mean		1.27						0.82						0.96					
SD		0.45						**0.62**						0.99					
Median		**1.3**						**0.62**						**0.81**					
Range	Min	**0.53**						**0.14**						0.57					
	Max	2.42						**2**						**2.12**					

*; significant (*p* ≤ 0.05) ns; non-significant (*p* > 0.05), rs=Spearman rank correlation coefficient

### Part II: Analysis of prediction accuracy for each facial region

**Fig. 6:**
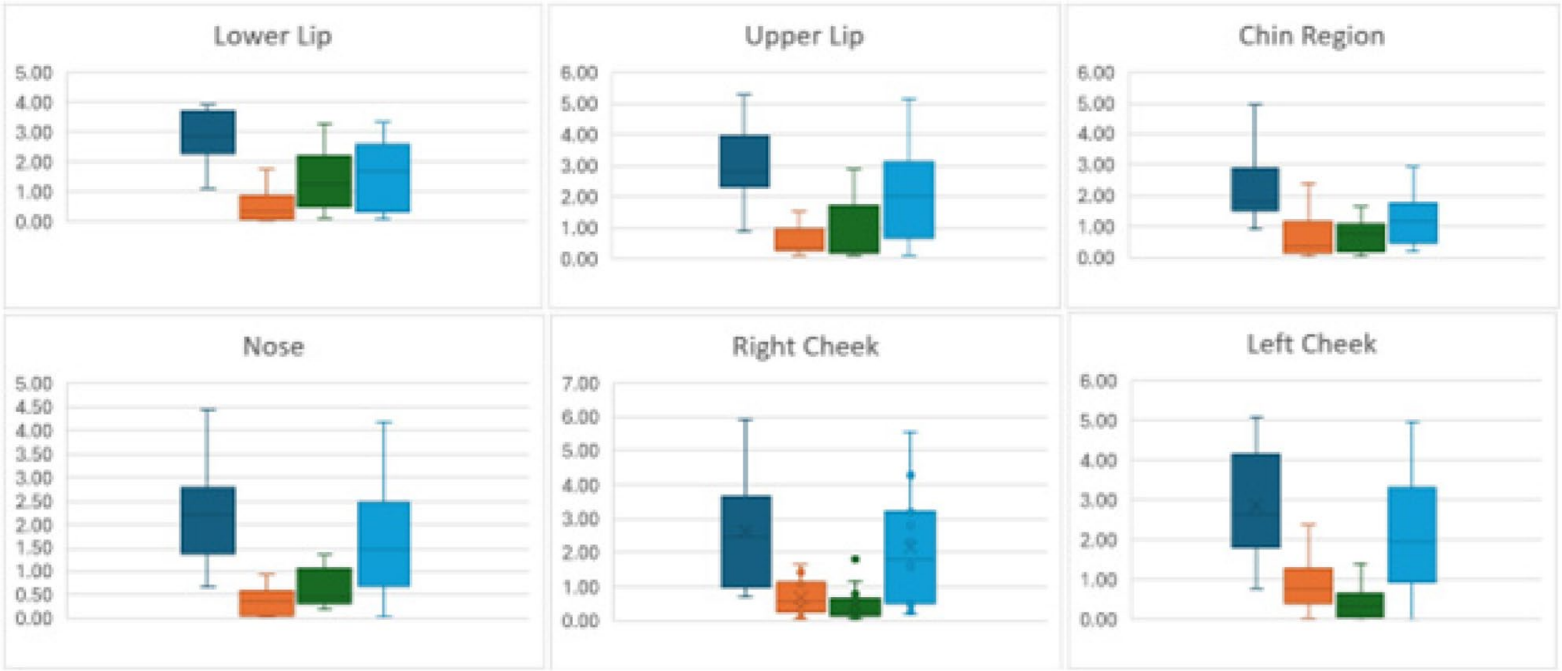
Box plots illustrating soft tissue prediction accuracy across facial regions in the X (orange), Y (green), and Z (light blue) dimensions, as well as the non-directional Euclidean distance (dark blue). The plots display the median, interquartile range, and whiskers representing the distribution of prediction errors

Differences between predicted and actual postoperative soft tissue changes were analyzed in the three spatial dimensions (x, y, and z) for each facial region (Fig. 6A–F; Table 2). Overall, the region-specific analysis demonstrated that prediction errors varied across facial aesthetic units, with the chin and nose showing the highest overall prediction accuracy, while the upper lip exhibited the greatest inaccuracy, particularly across the Z axis. A moderate variability was noted at the cheek regions. In all regions, the prediction errors were less across the X dimension. The color-coded deviation maps illustrated a general tendency toward mild underprediction of soft tissue changes following maxillary advancement and mandibular setback surgerys.

**Table 2:** Prediction errors between predicted and actual postoperative soft tissue surfaces across facial regions in the X (mediolateral), Y (vertical), and Z (anteroposterior) dimensions, and as overall Euclidean distances

Region	Direction	Median	Range	Min	Max	Q1	Q3	IQR
Upper lip	**Euclidean**	2.79	4.40	0.87	5.27	2.52	3.79	1.27
	**X**	0.37	1.39	0.15	1.53	0.30	0.94	0.63
	**Y**	0.27	2.77	0.11	2.88	0.25	1.71	1.47
	**Z**	2.04	5.06	0.11	5.17	0.74	3.00	2.27
Lower lip	**Euclidean**	2.86	2.81	1.10	3.92	2.31	3.70	1.39
	**X**	0.32	1.71	0.02	1.73	0.07	0.67	0.60
	**Y**	1.25	3.16	0.11	3.26	0.53	2.14	1.61
	**Z**	1.68	3.25	0.05	3.30	0.36	2.42	2.06
Chin	**Euclidean**	1.82	4.02	0.92	4.94	1.63	2.19	0.55
	**X**	0.37	2.84	0.01	2.85	0.16	0.69	0.53
	**Y**	0.76	2.88	0.05	2.93	0.31	0.99	0.68
	**Z**	1.16	2.74	0.20	2.94	0.50	1.63	1.13
Nose	**Euclidean**	2.19	3.77	0.67	4.44	1.44	2.70	1.26
	**X**	0.39	1.91	0.03	1.94	0.07	0.55	0.48
	**Y**	0.46	1.17	0.20	1.37	0.36	1.10	0.70
	**Z**	1.45	4.15	0.03	4.18	0.78	2.32	1.55
Right cheek	**Euclidean**	2.45	5.26	0.68	5.95	0.98	3.35	2.38
	**X**	0.57	1.55	0.08	1.63	0.31	1.09	0.78
	**Y**	0.22	1.74	0.08	1.82	0.13	0.49	0.36
	**Z**	1.78	5.36	0.19	5.54	0.55	3.20	2.65
Left Cheek	**Euclidean**	2.62	4.30	0.78	5.08	1.84	3.76	1.92
	**X**	0.78	2.33	0.04	2.37	0.63	1.14	0.52
	**Y**	0.37	1.40	0.01	1.41	0.10	0.46	0.36
	**Z**	1.97	4.95	0.00	4.96	1.18	3.11	1.93

### Part III: Analysis of prediction accuracy in each spatial dimension

**Fig. 7:**
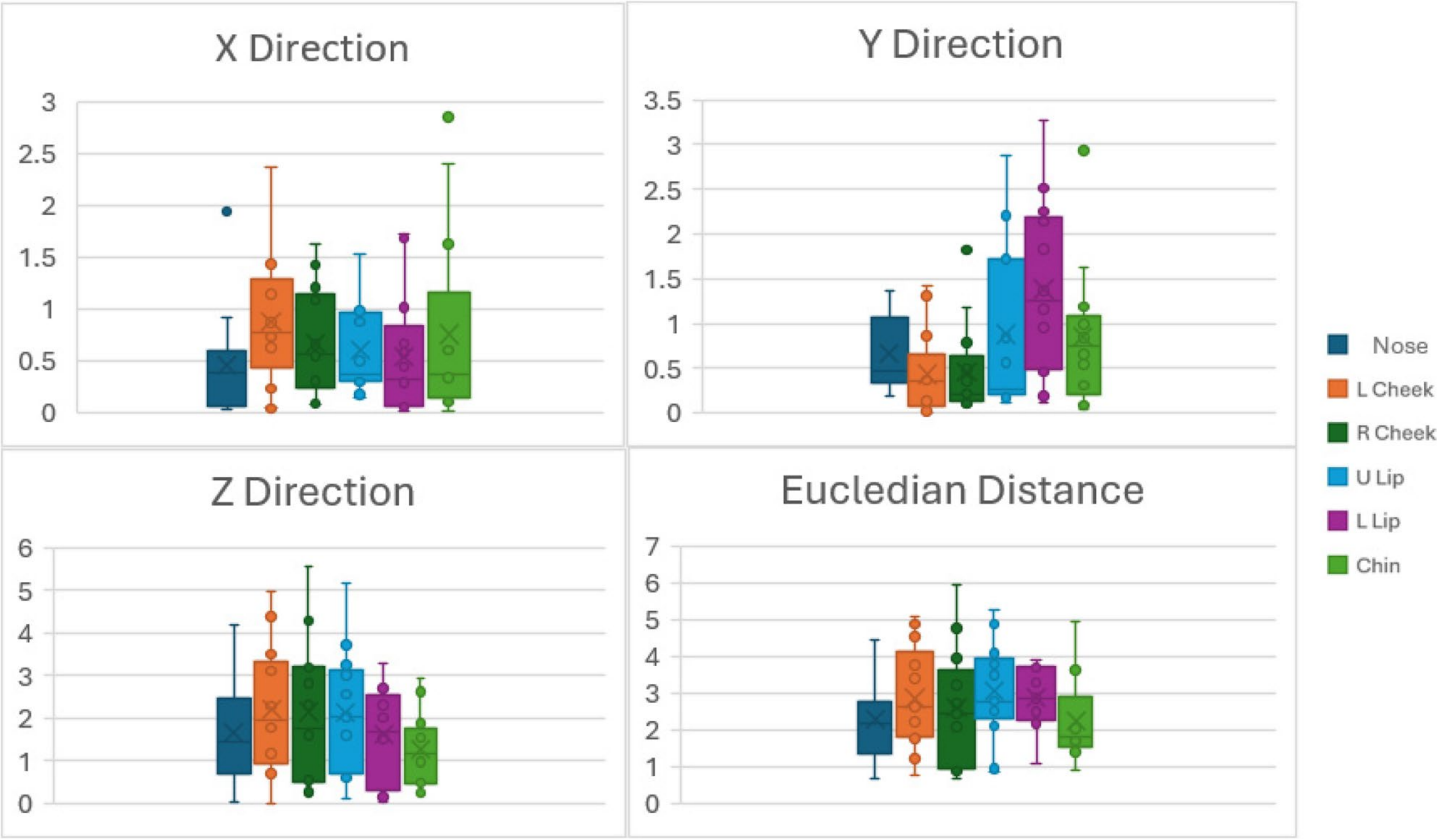
Box plots showing the distribution of simulation errors across the three spatial dimensions (X, Y, and Z)

Median prediction accuracy values were calculated for each spatial dimension across all facial regions. The upper lip demonstrated the largest prediction errors in the Z-direction, while the chin showed the lowest overall deviation (Table 3; Fig. 7). Underprediction of soft tissue changes was a universal finding in this study. This was not the case for the other two dimensions.

**Table 3:** Analysis of the prediction accuracy for each 3D dimension

Region	Nose	L cheek	*R* cheek	U Lip	L Lip	Chin	Overall
X Dimension	0.39	**0.78**	**0.57**	**0.37**	**0.32**	**0.37**	**0.38**
Y Dimension	0.46	**0.37**	**0.22**	**0.27**	**1.25**	**0.76**	**0.41**
Z Dimension	**1.45**	**1.97**	1.78	**2.04**	**1.68**	**1.16**	**1.73**
Euclidean Distance	**2.19**	**2.62**	**2.45**	**2.79**	**2.86**	**1.82**	**2.54**

## Discussion

This study evaluated the accuracy of soft-tissue prediction following bimaxillary orthognathic surgery using Dolphin 3D software in the Egyptian population. A comprehensive mathematical analysis was applied, with particular emphasis on the direction of soft tissue prediction errors across facial regions. This was achieved through conformation of a mathematical generic facial mesh and the application of dense surface correspondence analysis. The main novelty of the study is the conformation of generic facial mesh for the analysis of orthognathic soft tissue prediction accuracy in Egyptian population. The landmarks initiated the 3D mapping of the mesh on the face which was followed by computerized elastic deformations to “wrap” the mesh around the anatomical morphology of the face. We are not aware of similar studies in the English literature regarding the presented method on this particular ethnic group. The directionality of orthognathic soft tissue prediction has not been reported before. It was not our objective to compare different orthognathic software packages; rather, we present a universal, software-agnostic mathematical framework that may be extended to other prediction platforms in future comparative studies.

CBCT imaging was selected for analysis as it allows simultaneous visualization and quantification of skeletal movements and associated soft tissue changes. Although stereophotogrammetry provides high-resolution surface texture, it does not capture jaw skeletal structures and requires additional registration to CBCT scans, which may introduce inaccuracies. Despite limitations in soft tissue contrast and voxel size (0.3 mm), a standardized imaging protocol and voxel-based registration of the CBCT scans improved consistency and reproducibility [11–14]. Postoperative scans were obtained at 6–12 months to allow resolution of surgical edema, although longer follow-up may provide further insight into long-term stability.

Previous investigations have largely relied on a limited number of anatomical landmarks for facial analysis [15–19]. The landmark-based assessment does not adequately represent complex three-dimensional facial topography. Surface-based approaches using thousands of mesh points provide a more comprehensive description [20, 21]; however, generic meshes lack true anatomical correspondence between vertices. Furthermore, superimposition of corresponding surface meshes using the Iterative Closest Point (ICP) algorithm includes unaffected regions, potentially underestimating actual morphological change [22]. It also does not guarantee accurate superimposition of the corresponding anatomical structures Iterative closest points appraoch does not guarantee structural correspondence for the accurate analysis 3D surface differences.

On the other hand, the conformation mesh has enabled accurate vertex-to-vertex correspondence and eliminated key limitations of ICP-based analysis, allowing robust quantification of regional and directional prediction errors with sub-millimeter resolution. It is important to note that dense correspondence analysis has been used previously [23]. Peter Claes et al. described a partially dense set of 3D points connected to form a wireframe of polygons representing the full 3D facial structure. As explained by the authors, a set of quasi-landmarks was generated for dense correspondence analysis. However, these quasi-landmarks lost compatibility with the original configuration when assessing corresponding 3D facial images, necessitating further re-identification prior to analysis. Although Euclidean distances between quasi-landmarks were measured, the directionality of the disparities between the superimposed surfaces were not considered.

In the present study, the measured disparities between superimposed corresponding surfaces were stratified along the x, y, and z axes. To the best of our knowledge, this approach has not been previously reported in the evaluation of orthognathic surgical planning accuracy. The directional analysis of these distances provides unprecedented insight into vertical, anteroposterior, and mediolateral errors in soft tissue prediction for the correction of dentofacial deformities.

Subdivision into anatomical regions has been considered previously. Hou et al. reported that anatomical region based analysis improved the understanding of soft tissue prediction in orthognathic surgery [24], while Ruggiero et al. developed a patient-specific numerical model using finite element analysis [25]. However, these approaches did not resolve the limitation whihc is the lacking of anatomical correspondence in surface analysis. The study applied Finite Element Analysis to investigate the anatomical effect of single muscles in specific areas of the midface [25]. The use of conformation mesh in our study facilitated the subdivision of the face into aesthetic anatomical regions which has not been used before for the assessment of orthognathic soft tissue prediction accuracy.

Gonzalez-Ulloa introduced the concept of facial aesthetic units without clinical relevance [26]. In our study, these units were refined to establish clinically and statistically relevant divisions, enabling systematic regional analysis and a more objective assessment of facial morphology. Directional analysis in the x, y, and z dimensions provides insight into the trajectories of prediction inaccuracies, which is particularly relevant in correcting facial asymmetry.

Color-coded error maps used in our study provided intuitive visualization of prediction accuracy, facilitating identification of high-error regions. This aligns with recommendations by Resnick et al. emphasizing the importance of the visual tools for understanding predictive limitations in orthognathic surgery [6] and supports a clinically meaningful advancement in soft tissue prediction analysis and planning.

Olejnik et al. highlighted that landmark identification errors significantly impact on orthognathic predictive accuracy [27]. In the present study, the highest landmarking errors were observed in mediolateral and vertical dimensions. The measured average landmarks prediction error of 1.44 ± 0.63 mm was acceptable clinically [6].

Several studies have reported variable accuracy of three-dimensional soft tissue prediction software [28, 29]. Resnick et al. identified limitations of Dolphin 3D software package, particularly in lateral regions [6], while Knoops et al. reported underprediction in paranasal regions with larger maxillary advancements [29]. A systematic review by Olejnik et al. confirmed greater inaccuracies in orthognathic prediction of the lateral facial regions [27]. Differences in analytical methods influence the reported results [30]. While the framework provides methodological insight into spatial prediction pattern, its clinical relevance lies in identifying the facial regions of consistent accuracy versus areas which requiring caution in orthognathic planning.

Region-specific analysis demonstrated that the upper lip exhibited the greatest anteroposterior discrepancy. This likely due to its complex morphology and variability in elasticity and muscle tone. The Dolphin 3D algorithm is based on photographic morphing techniques extrapolated from two-dimensional models, relying on landmark displacement and interpolation rather than true biomechanical modeling. Consequently, curvature changes and soft tissue sliding cannot be fully reproduced, explaining the observed Z-axis underprediction. More recent approaches incorporating finite element modeling and machine learning aim to address these limitations by accounting for tissue biomechanics and nonlinear deformation. Integration of such models into future planning software may reduce region-specific inaccuracies.

The lower lip and chin demonstrated relatively high predictive accuracy, likely due to closer biomechanical coupling with the mandible. Consistent with previous reports, nasal prediction remains limited. In this cohort, lateral regions such as the cheeks showed comparable accuracy to midline regions. The discrepancies in orthognathic preiction planning are region- and direction-specific rather than strictly anatomical [4].

These discrepancies should be interpreted within the context of clinical acceptability. A discrepancy of approximately 2.0 mm is generally considered clinically acceptable and unlikely to influence treatment planning [3, 6, 27]. In our study, upper-lip anteroposterior errors approached this threshold, indicating underprediction of the soft tissue changes with maxillary advancement. Clinicians should therefore interpret upper-lip predictions cautiously and anticipate slightly greater advancement when planning maxillary movements.

Dimensional analysis showed minimal errors in the X and Y axes, indicating adequate prediction of mediolateral and vertical changes. In contrast, the Z-dimension demonstrated consistent underprediction, particularly in the upper lip and cheeks. Similar findings were reported by Khambay et al. [7]. Color maps confirmed a general trend of underprediction, highlighting the need for algorithmic refinement of the orthognathic software packages [30]. Incorporation of patient-specific biomechanics or machine-learning approaches may improve prediction accuracy.

Although justified by power calculation, the limited cohort (*n* = 13), this restricts generalizability and the findings should be considered exploratory. The results of the study should be interpreted cautiously due to the small sample size of one ethnic group. Larger prospective multicenter studies with diverse populations are recommended. Future research should explore the relationship between the magnitude of the surgical movement and regional prediction accuracy, facilitating the development of direction-specific biomechanical or data-driven models. Collaboration between clinicians and computational scientists will be essential to advance orthognathic planning software and improve patient care. Future studies may apply this framework prospectively to compare multiple platforms, enabling standardized cross-software validation of orthognathic soft tissue prediction accuracy.

## Conclusion

Dolphin software demonstrated clinically acceptable accuracy for soft-tissue prediction following bimaxillary orthognathic surgery; however, this was varied across facial regions and spatial dimensions. Consistent underprediction was observed in the upper lip, particularly in the anteroposterior direction, and modest inaccuracies were noted in lateral facial regions. The application of dense surface correspondence, generic mesh conformation, and direction-specific analysis provided a detailed understanding of the prediction patterns at all facial regions and across the three dimensions. These findings support the clinical utility of three-dimensional prediction planning while underscoring the need for cautious interpretation in certain regions with complex soft-tissue morphology and anatomical relations, particularly when planning Le Fort I maxillary advancement sagittal split mandibular setback surgery. Further multicenter studies are recommended which should other ethnic groups.

## Data Availability

The datasets generated and analyzed during the current study are available from the corresponding author upon reasonable request, and segmentation into facial aesthetic units has provided a more precise assessment of soft tissue changes in all.
